# Editorial: Managing COVID-19 in heart and lung transplantation: clinical challenges and emerging solutions

**DOI:** 10.3389/fsurg.2026.1843253

**Published:** 2026-04-17

**Authors:** Chengliang Yang, Wenjun Mao, Ömer Senbaklavaci

**Affiliations:** 1 Prevention of Organ Failure (PROOF) Centre of Excellence, St. Paul’s Hospital, Vancouver, BC, Canada; 2 Centre for Heart Lung Innovation, University of British Columbia, Vancouver, BC, Canada; 3 Department of Medicine, University of British Columbia, Vancouver, BC, Canada; 4 Providence Research, Providence Health Care, Vancouver, BC, Canada; 5 Department of Cardiothoracic Surgery, The Affiliated Wuxi People’s Hospital of Nanjing Medical University, Wuxi People’s Hospital, Wuxi Medical Center, Nanjing Medical University, Wuxi, China; 6 Department of Thoracic Surgery, University Hospital Bern, Bern, Switzerland

**Keywords:** COVID-19, heart transplantation, immunosuppression, long COVID, lung transplantation

In the post-pandemic era, COVID-19 continues to pose an important clinical challenge in heart and lung transplantation, affecting infection risk, perioperative management, immunosuppressive strategies, and long-term outcomes. Although advances in vaccination, antiviral therapies, and supportive care have improved outcomes in the general population, heart and lung (cardiothoracic) transplant recipients remain vulnerable due to chronic immunosuppression, high comorbidity burden, and complex pharmacologic management. This increased vulnerability may also be partly explained by impaired humoral and cellular immune responses to SARS-CoV-2 vaccination and the effects of maintenance immunosuppression on infection severity reported in transplant recipients (1, 2). Beyond the acute phase, the long-term consequences of COVID-19, including long COVID and its impact on transplant outcomes, are still incompletely understood. These challenges underscore the need for focused research addressing the unique risks and management considerations in this population.

This Research Topic was designed to explore the multifaceted impact of COVID-19 on cardiothoracic transplant recipients, including clinical outcomes, long-term complications, and therapeutic management strategies. The six articles in this collection converge on three key themes: the persistent clinical vulnerability of heart and lung transplant recipients, the emerging recognition of long-term post-infection sequelae, and the complexity of pharmacologic management in immunocompromised patients.

Three original research articles in this collection describe the clinical outcomes of COVID-19 among heart and lung transplant recipients. In a large single-center cohort of heart transplant recipients, Zhang et al. identified advanced age, diabetes, and chronic kidney disease as independent risk factors for severe COVID-19 infection, while age and chronic kidney disease were also associated with increased mortality. COVID-19 vaccination was associated with reduced mortality (Zhang et al.). Consistent with this finding, recent Infectious Diseases Society of America (IDSA) guidelines also recommend COVID-19 vaccination for immunocompromised populations, including solid organ transplant recipients, based on evidence demonstrating reduced risks of severe disease and mortality (3). The study also identified sirolimus use as a potential risk factor for severe infection, demonstrating the complexity of immunosuppressive management during viral illness (Zhang et al.). This is further supported by a large national French cohort study of over 60,000 recipients, where sirolimus was associated with increased COVID-19 hospitalization risk in heart transplant recipients (2). These findings suggest that mammalian target of rapamycin (mTOR) inhibitors such as sirolimus may influence SARS-CoV-2 outcomes differently from other maintenance regimens, supporting a more individualized approach to immunosuppression adjustment in infected heart transplant recipients. In a single-center retrospective case-control study of lung transplant recipients, Hanna et al. found that SARS-CoV-2 infection was associated with an increased risk of all-cause mortality compared with matched controls. However, the study did not observe significant declines in pulmonary function or increased rates of acute cellular rejection, suggesting variable long-term graft outcomes following COVID-19 infection (Hanna et al.). A third study of lung transplant recipients further highlighted the high burden of SARS-CoV-2 infection within this cohort and substantial mortality among patients with severe disease. Zhang et al. also found older age and pre-existing chronic lung allograft dysfunction as risk factors for severe COVID-19, underscoring the importance of identifying higher-risk patients in this vulnerable population (Zhang et al.). Together, these studies highlight the persistent risk of severe COVID-19 in cardiothoracic transplant recipients and the need for improved risk stratification and longitudinal monitoring strategies. These observations also raise an important question for transplant programs: how should transplant centers balance infection prevention, immunosuppression management, and long-term graft monitoring in the post-COVID era?

Beyond acute infection, the long-term consequences of COVID-19 are increasingly recognized as a major clinical concern. Long COVID represents a major public health challenge with substantial long-term clinical and socioeconomic consequences (4). Emerging evidence also indicates the disproportionate burden of COVID-19 and its long-term sequelae among immunocompromised populations, including transplant recipients. Within this collection, Sandoval et al. examined long COVID in solid organ transplant recipients and reported that approximately 5% of patients were diagnosed with long COVID. Patients with long COVID were generally older, had a higher comorbidity burden, and were more frequently cardiothoracic (heart and lung) transplant recipients compared with those without long COVID. Notably, long COVID was not significantly associated with graft failure or mortality in this cohort, highlighting the complexity of defining clinically meaningful long-term outcomes in transplant populations (Sandoval et al.). National COVID Cohort Collaborative analyses have demonstrated that solid organ transplant recipients are at increased risk of post-acute sequelae compared with non-immunocompromised populations (5). However, findings from this Research Topic suggest that the presence of post-COVID complications may not necessarily correspond to worse graft outcomes (Sandoval et al.), underscoring the need for more nuanced clinical risk assessment despite the recognized burden of post-acute sequelae in transplant populations. Collectively, these observations indicate that while long COVID may substantially affect quality of life and functional status, its relationship with traditional transplant outcomes remains uncertain and warrants prospective investigation. Beyond the studies included in this Research Topic, pathophysiological models have proposed that persistent SARS-CoV-2 reservoirs and chronic immune dysregulation may contribute to long COVID pathogenesis, particularly in immunocompromised populations (6). Emerging biomarker research supports the need for validated molecular and cellular markers to improve diagnostic precision, define biological phenotypes, and enable risk stratification in long COVID (7).

In addition to clinical outcomes, this Research Topic demonstrates important therapeutic challenges related to COVID-19 management in immunocompromised populations. Two case reports show clinically significant drug-drug interactions between COVID-19 antiviral therapies and complex pharmacologic regimens frequently encountered in transplant medicine. López-Hernández et al. described an unexpected increase in voriconazole plasma concentrations associated with nirmatrelvir/ritonavir therapy, resulting in drug toxicity and treatment interruption. This case demonstrates the importance of therapeutic drug monitoring and careful assessment of drug-drug interactions when managing complex medication regimens (López-Hernández et al.). Similarly, Xiong et al. reported markedly supratherapeutic tacrolimus concentrations following nirmatrelvir/ritonavir exposure in a lung transplant recipient who self-administered antiviral therapy without appropriate dose adjustment. This case demonstrates the risk of tacrolimus toxicity due to clinically significant drug-drug interactions between tacrolimus and nirmatrelvir/ritonavir (Xiong et al.). Careful immunosuppressive management, therapeutic drug monitoring, and patient education are critical for transplant recipients receiving complex immunosuppressive regimens. Together, these reports underscore the critical role of multidisciplinary transplant care teams, including clinical pharmacists, in mitigating drug interaction risks and improving patient safety. These therapeutic complexities may also complicate the longitudinal assessment of post-COVID sequelae, where overlapping medication effects and persistent symptoms can challenge clinical interpretation.

Across these studies, three practical lessons and research priorities for transplant care emerge. First, despite advances in prevention and treatment, cardiothoracic transplant recipients remain at increased risk for severe COVID-19 and require continued vigilance. Second, the long-term consequences of COVID-19 represent an emerging challenge extending beyond traditional transplant outcomes such as graft survival and mortality. Third, the complexity of managing COVID-19 in transplant recipients underscores the importance of individualized therapeutic strategies, particularly in the context of polypharmacy and drug interaction risks.

Future research should focus on improving risk stratification strategies and identifying patients at highest risk for severe infection and long-term complications. Prospective studies examining the long-term impact of COVID-19 on graft function, functional status, and patient-reported outcomes are needed. Clarifying interactions between immunosuppressive strategies and emerging antiviral therapies also remains a priority.

In conclusion, the studies presented in this Research Topic provide valuable insights into the ongoing challenges posed by COVID-19 in heart and lung transplant recipients. Despite recent advances, significant knowledge gaps remain regarding long-term outcomes, optimal management strategies, and prevention of treatment-related complications. Continued collaborative research and multidisciplinary clinical innovation will be important for improving the care and long-term outcomes of transplant recipients. We hope this Research Topic stimulates further investigation to address these remaining challenges.
